# Systematic review of classification systems for locally recurrent rectal cancer

**DOI:** 10.1093/bjsopen/zrab024

**Published:** 2021-04-08

**Authors:** Z Rokan, C Simillis, C Kontovounisios, B J Moran, P Tekkis, G Brown

**Affiliations:** 1 Department of Radiology, Royal Marsden Hospital, London, UK; 2 Pelican Cancer Foundation, Basingstoke, UK; 3 Cambridge Colorectal Unit, Addenbrookes Hospital, Cambridge, UK; 4 Department of Surgery & Cancer, Imperial College, London, UK; 5 Department of Colorectal Surgery, Royal Marsden Hospital, London, UK; 6 Department of Colorectal Surgery, Chelsea & Westminster Hospital, London, UK; 7 Department of Peritoneal Malignancy, Basingstoke & North Hampshire Hospital, Basingstoke, UK

## Abstract

**Background:**

Classification of pelvic local recurrence (LR) after surgery for primary rectal cancer is not currently standardized and optimal imaging is required to categorize anatomical site and plan treatment in patients with LR. The aim of this review was to evaluate the systems used to classify locally recurrent rectal cancer (LRRC) and the relevant published outcomes.

**Methods:**

A systematic review of the literature prior to April 2020 was performed through electronic searches of the Science Citation Index Expanded, EMBASE, MEDLINE and CENTRAL databases. The primary outcome was to review the classifications currently in use; the secondary outcome was the extraction of relevant information provided by these classification systems including prognosis, anatomy and prediction of R0 after surgery.

**Results:**

A total of 21 out of 58 eligible studies, classifying LR in 2086 patients, were reviewed. Studies used at least one of the following eight classification systems proposed by institutions or institutional groups (Mayo Clinic, Memorial Sloan-Kettering – original and modified, Royal Marsden and Leeds) or authors (Yamada, Hruby and Kusters). Negative survival outcomes were associated with increased pelvic fixity, associated symptoms of LR, lateral compared with central LR and involvement of three or more pelvic compartments. A total of seven studies used MRI with specifically defined anatomical compartments to classify LR.

**Conclusion:**

This review highlights the various imaging systems in use to classify LRRC and some of the prognostic indicators for survival and oncological clearance based on these systems. Implementation of an agreed classification system to document pelvic LR consistently should provide more detailed information on anatomical site of recurrence, burden of disease and standards for comparative outcome assessment.

## Introduction

Rectal cancer remains a globally significant problem, with approximately 8000–9000 new patients diagnosed each year in the UK^1^. Surgical resection is still the best chance of cure for patients with resectable rectal cancer, however, despite the introduction of selective neoadjuvant chemoradiotherapy and ‘watch-and-wait’ strategies, local recurrence (LR) rates remain between 5 and 18 per cent^2–4^. This is a significant issue and it can lead to significant morbidity, with symptoms including persistent pain, tenesmus, malodourous discharge and bleeding, ultimately resulting in death^2^^,^^5^.

Development of surgical techniques, including resection beyond total mesorectal excision (TME) and pelvic exenteration, in conjunction with chemoradiotherapy, have revolutionized the treatment of patients with locally recurrent rectal cancer (LRRC). Radical resection can achieve complete oncological clearance (R0) in 55 per cent of patients^2^. Reported survival rates following R0 resection of LRRC indicates a 3-year disease-free survival to be approximately 57 per cent^2^ with 3-year overall survival between 48 and 65 per cent^2^^,^^4^. This complex, often multivisceral surgery, may also significantly impact a patient’s quality of life^6^, so careful use of imaging for treatment planning is crucial.

Treatment is predominantly guided by MRI in combination with CT and clinical examination. Currently there is no single imaging system classifying LRRC, which has been validated against survival and oncological outcomes, although multiple anatomical and operative classification systems have been proposed^7–15^. As a result, patient selection and information on selection methodology, neoadjuvant treatment and surgical planning are largely heterogeneous between centres.

The aim of this study was to review the most frequently used classification systems in describing LRRC and quantify the prognostic information provided by each system, with respect to the outcome measures described below.

## Methods

### Search strategy

This systematic review was based on a written protocol and was reported in line with Preferred Reporting Items for Systematic Reviews and Meta-Analyses (PRISMA)^16^ and Assessing the Methodological Quality of Systematic Reviews (AMSTAR) guidelines^17^. A comprehensive literature search was performed using a combination of free-text terms and controlled vocabulary of the following databases: PubMed MEDLINE, Embase, Science Citation Index Expanded, and Cochrane Central Register of Controlled Trials (CENTRAL) in The Cochrane Library. The detailed search strategy is provided in *TableS1*.

All abstracts, studies and citations identified were reviewed, and the references of the identified studies were also searched. No restrictions were made based on language, publication year, or publication status. The literature search was complete up to 28 April 2020.

### Selection criteria

Prospective and retrospective studies were considered for this systematic review if studies met the following criteria:

- Reported on patients with LRRC or rectosigmoid cancer who underwent previous ‘curative’ surgery.
- Reported on patients where the anatomical location of LR or a defined classification system for describing LRRC was documented.

### Outcome of interest

The primary outcome was to evaluate which classification systems have been previously or are currently being used to describe the location of a locally recurrent tumour within the pelvis, following surgery for primary rectal/rectosigmoid adenocarcinoma. The secondary outcome was to assess the relevant information provided by these classification systems with respect to prognostic/survival information and prediction of R0 resection. Two review authors independently determined the eligibility of all retrieved studies and extracted the required data from the included studies.

## Results

### Studies

**Fig. 1:**
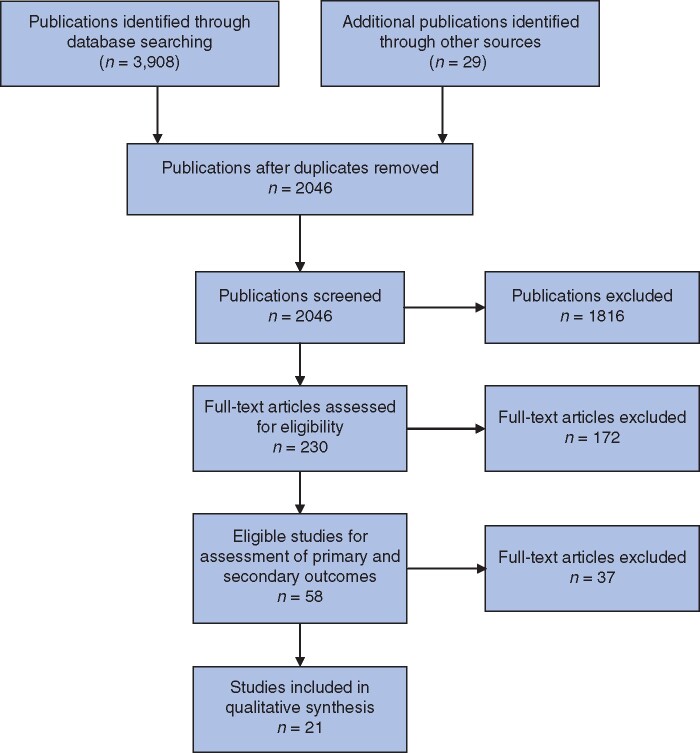
Study flow diagram

A total of 3908 references were identified through systematic electronic searches of Science Citation Index Expanded (1140 references), EMBASE (1091), MEDLINE (1563) and CENTRAL (114). A further 29 studies were identified from the references of the above studies. There were 1891 duplicates between databases and duplicates were excluded. A further 1816 clearly irrelevant references were excluded through screening titles and reading abstracts. The remaining 230 studies were investigated in full-text detail and a further 172 studies were excluded. *Figure 1* shows the study flow diagram. Fifty-eight cohort studies fulfilled the inclusion criteria of this systematic review^7^^,^^8^^,^^10^^,^^12–15^^,^^18–68^. Of these, thirty-seven did not classify LR according to a defined system and were therefore included in the primary outcome assessment but excluded from secondary outcome analysis. The remaining 21 studies constituted the basis of this review and characteristics of patients within these studies, including demographic information, primary tumour staging, treatment received and relevant outcomes, are summarized in *Table 1*.

**Table 1:** Demographic and tumour information with relevant outcomes in studies using defined systems to classify LRRC

Study	Country	Years of study	Type of cohort study	Mean or median age (years)	No. of patients followed/included in study	**Male : female (TP or R)**	Stage of primary tumours (TP or R)	Lymph node status (TP or R)	Perioperative treatment of primary tumour	Operation performed for primary cancer leading to recurrence (TP or R)	No. of local recurrences included	Imaging used to diagnose recurrence	Classification system used	Recurrences classified	Relevant outcomes highlighted
Bird *et al*. 2018^22^	Australia	19	Prospective	(R): 63	98	(R): 61M : 37F	TNM (R): T1–T2 N0 = 14 T3N0 = 26 T2N+ve = 2 T3N+ve = 21 T4N0 = 9 T4N+ = 6 Unknown = 20	(R): N-ve = 49 N+ve = 29 Unknown = 20	(R): Adjuvant chemotherapy: Yes = 45 No = 48 Unknown = 5 Chemoradiation: Neoadjuvant = 34 Adjuvant = 6 None = 57 Unknown = 1	(R): Sphincter-preserving = 79 Non-sphincter-preserving = 19	98	MRI/CT/PET-CT	Yamada *et al.*^8^ and regional anatomical	Localized = 9 Sacral invasive = 6 Lateral invasive = 15 Unknown = 9 Anastomosis only = 30 Presacrum =24 Genitourinary = 24 Lateral = 14 Lymph nodes = 1 Unknown = 9	Poorer PFS in patients with sacral and lateral invasive LR (*P* < 0.05)
Boyle *et al*. 2005^12^	UK	7	Retrospective	(R): 56	64	(R): 38M : 26F	Dukes (R): A = 8 B = 19 C = 26 Unknown = 11	Not reported	Not reported	(R): AR = 35 APER = 22 Proctectomy with end colostomy = 5 Total colectomy and end ileostomy = 1 Resection of rectal stump = 1	64	MRI/PET	Regional anatomical (Leeds group)^21^ and Symptoms and fixity (Mayo clinic)^7^	Central = 23 Sacral = 10 Sidewall = 21 Composite = 10 F0 = 22 F1 = 28 F2 = 14	37.0% of patients with F0/F1 LR suffered postoperative complications compared with 54.5% in those with F2 disease
Hahnloser *et al*. 2003^30^	USA	15	Retrospective	(R): 60.8	304	(TP): 192M : 112F	Astler-Coller (R): A = 15 B1 = 54 B2 = 76 B3 = 15 C1 = 19 C2 = 72 C3 = 15 Unknown= 38	(R): N-ve = 160 N+ve = 106 Unknown = 38	Not reported	(R): Sphincter-preserving = 200 Stoma = 104	304	CT/MRI	Symptoms and fixity (Mayo Clinic)^7^	F0 = 103 F1 = 84 F2 = 66 F3 = 51	Complication rates significantly associated no. of sites of fixation of the LR: 20% in F0/F1 tumours, 35% in F2 tumours and 32% in F3+ tumours (*P* = 0.05). Increasing the number of points of pelvic fixation significantly reduced survival at both 3 and 5 years (*P* < 0.0001)
Hruby *et al*. 2003^13^	NZ	13	Retrospective	(R): 70	269	(R): 150M : 119F	TNM (R): T1 = 4 T2 = 29 T3 = 198 T4 = 23 Unknown = 15	(R): N-ve = 129 N+ve = 140	(R): No radiotherapy = 269 Adjuvant chemotherapy = 36	(R): APER = 100 LE = 8 LAR = 154 Other = 6 Unknown = 1	268	Not stated	Regional anatomical (Hruby et al.)^13^	Anterior central = 29 Posterior central = 127 Sidewall = 30 Anastomosis = 57 Perineum = 15 Perineum and pelvis = 9 Unknown = 1	Primary T4 rectal cancers most frequently recurred in the anterior central compartment (*P* < 0.01) and perineal LR occurred following an APER (*P* < 0.01)
Iversen *et al*. 2018^32^	Sweden	10	Retrospective	(R): 65	95	(R): 59M : 36F	Not reported	Not reported	(R): Neoadjuvant radiotherapy = 54	Primary operative procedure not stated	184	MRI	Regional anatomical (Memorial Sloan-Kettering)^10^	Lateral = 46 Axial = 67 Anterior = 40 Posterior = 31	Rate of R0 resection greater if lateral compartment not involved intraoperatively in comparison with patients with an involved lateral compartment (90 versus 63%, *P* = 0.004)
Kanemitsu *et al*. 2010^33^	Japan	25	Retrospective	(R): 57	101	(R): 57M : 44F	Dukes (R): A = 18 B = 21 C = 52 D = 5 Unknown = 5	(R): N-ve = 39 N+ve = 52 Unknown = 10	(R): Adjuvant treatment: Chemotherapy = 33 Radiotherapy = 3	(R): LE = 4 HAR = 15 LAR = 46 APR = 32 Hartmann's = 4	101	CT/MRI	Regional anatomical Modified Yamada *et al*.^8^	Anastomotic = 18 Visceral/lower sacral invasive = 41 Upper sacral/lateral invasive = 27 Unknown = 15	Pattern of pelvic invasion affected likelihood of R0 resection (*P* = 0.005) and local DFS following surgery for LR (*P* = 0.028)
Kusters M *et al*. 2009^14^	Japan * and The Netherlands	9	Retrospective	(TP): 58	324	(TP): 215M : 109F	TNM (TP): pT1 = 52 pT2 = 107 pT3 = 160 pT4 = 5	(TP): pN0 = 192 pN1 = 80 pN2 = 52	(TP): Neoadjuvant therapy = 0 Adjuvant therapy: Radiotherapy = 5 Chemotherapy = 23 None = 297	(TP): APER = 113 Hartmann’s = 3 LAR = 195 PE = 13 LLND: Standard TME = 134 Unilateral LLND = 69 Bilateral LLND = 121	23	Not stated	Regional anatomical (Kusters *et al*.^39^ and Roels^71^)	Presacral = 2 Lateral = 8 Anterior = 1 Anastomosis = 5 Perineum = 5 Unknown = 2	N/A
Kusters *et al.* 2009^14^	The Netherlands	12	Prospective	(TP): 63	290 (247 with previous R0 resection)	(TP): 179M : 111F	TNM (TP): cT3+ = 113 cT4 = 177	Not reported	(TP): Neoadjuvant treatment: RT only = 86 Chemoradiotherapy = 204 IORT = 290 Adjuvant chemotherapy = 39	(TP): APER = 138 Abdominotranssacral resection = 12 LAR = 132 PE = 8	Out of 247 patients with R0 resection: 18	Not stated	Regional anatomical (Kusters *et al*.)^39^	Presacral = 8 Posterolateral = 1 Lateral = 2 Anterior = 4 Anastomotic= 1 Perineal = 2	N/A
Kusters *et al.* 2010^38^	The Netherlands	Not stated	Prospective	(R): 65	1417	(R): 69M : 45F	TNM (R): pT2 = 15 pT3 = 90 pT4 = 9	(R): pN0 = 29 pN1 = 46 pN2 = 39	(R): Neoadjuvant radiotherapy: Yes = 36 No = 78	(R): APER = 47 Hartmann = 6 LAR = 61	114	Not stated	Regional anatomical (Kusters *et al*.)^39^	Presacral = 40 Lateral = 23 Anterior = 20 Anastomosis = 24 Perineum = 4 Unknown = 3	TME with radiotherapy for primary rectal adenocarcinoma had a 5-year LR rate of 0.7% in the anterior compartment compared with 2.7% in those patients undergoing TME surgery alone (*P* = 0.003). APER for primary rectal adenocarcinoma had a 5-year LR rate of 11.7%, usually occurring in the presacral compartment (45%), compared with a 5-year LR rate of 7.8% in those undergoing LAR which usually resulted in anastomotic (36%) and presacral (28%) LR
Moore *et al*. 2004^10^	USA	6	Retrospective	(TP): 59	119 (101 pelvic recurrence of rectal cancer, 18 pelvic recurrence of colon cancer)	(TP) 64M : 55F	TNM (TP): ** T0–2 = 37 T3–4 = 71	(TP): ** N0–X = 66 N1–2 = 46	(TP): Adjuvant radiotherapy +/- chemotherapy = 59	(R): In 101 pelvic recurrence of rectal cancer: APER = 15 LAR = 77 TAE = 8 Kraske = 1	In 101 pelvic recurrences of rectal cancer: 174	CT/MRI	Regional anatomical (Memorial Sloan-Kettering^10^)	Axial = 38 Lateral= 47 Anterior = 47 Posterior = 42	If pelvic sidewall not involved by recurrent tumour on imaging – R0 resection in 60% of patients. When axial compartment alone occupied by tumour intraoperatively – R0 resection rate of 70% compared with 43% when other compartments were involved (*P* < 0.001). When both the axial and anterior compartments occupied by recurrent tumour – R0 resection in 72% compared with 42% when tumour occupied other intrapelvic compartments (*P* = 0.003) Iliac vessel involvement – R0 resection in 17% compared with 55% when not involved (*P* = 0.01)
Pilipshen *et al*. 1984^15^	USA	8	Prospective	(TP): 62	412	(TP): 243M : 169F	Dukes (R): A = 18 B = 32 C = 55	(R): N-ve = 50 N+ve = 55	(TP): Neoadjuvant irradiation = 113 Adjuvant irradiation = 17 (R): Neoadjuvant irradiation = 33	(R): APER = 39 LAR = 66	105	Not stated	Regional anatomical +/- fixation (Previous Memorial Sloan Kettering^50^)	Anastomotic = 9 Perianastomotic = 3 Pelvic with fixation = 54 Pelvic without fixation = 7 Pelvic with anastomotic (with or without fixation) = 32	N/A
Roodbeen *et al*. 2020^67^	The Netherlands	7	Retrospective	(TP): 64	767	(R): 21M : 3F	TNM (TP): T1 = 23 T2 = 196 T3 = 421 T4 = 52 Tx = 4 Unknown = 71	(TP): N0 = 214 N1 = 303 N2 = 175 Nx = 4 Unknown = 71	(R): Previous neoadjuvant treatment = 17 Unknown = 7	(TP): TaTME: APER/ELAPE = 91 Hartmann's = 5 LAR = 659 Proctocolectomy = 12	24	Not stated	Regional anatomical (Royal Marsden group^11^)	Lateral = 10 Central = 6 Posterior = 8	N/A
Sinaei *et al*. 2013^51^	Canada	11	Retrospective	(R): 61	42	(R): 26M : 16F	Not reported	Not reported	Not reported	(R): APER = 16 Rectal anastomosis = 26	65	MRI	Regional anatomical (Memorial Sloan-Kettering^10^) and anatomical	Axial = 19 Lateral = 6 Anterior = 14 Posterior = 13 Other = 13 (pelvic floor = 7, sciatic nerve = 2, obturator nerve = 1, perineum = 1, abdominal wall = 1, adnexa = 1)	N/A
Suzuki *et al*. 1996^7^	USA	7	Prospective	(R): 62.9	65	(R): 38M : 27F	Astler-Coller (R): A = 6 B1 = 18 B2 = 11 B3 = 1 C1 = 4 C2 = 14 C3 = 3 Unknown = 8	(R): N-ve = 36 N+ve = 21 Unknown = 8	Not reported	(R): LAR = 34 APER = 15 Local excision = 15 Hartmann’s = 1	65	CT	Symptoms and fixity (Mayo clinic^7^)	F0 = 43 F1 = 13 F2 = 8 F3 = 1	Following surgery for LRRC increasing risk of severe complications as the degree of fixation increased, from 14% in F0 patients, to 44% in F3 patients The 3- and 5-year survival rates were 68.4 and 37.3% respectively for patients without pain (S0/S1), compared with 31.6 and 26.3% respectively for those with pain (S2). The 3- and 5-year survival rates were 61.3 and 50% respectively for those patients with no disease fixation (F0) and those with some degree of disease fixation (F1–3)
Uehara *et al*. 2015^53^	Japan	7	Retrospective	(R): 66	35	(R): 27M : 8F	UICC (R): I = 5 II = 15 III = 14 IV = 1	(R): N-ve = 20 N+ve = 14 Unknown = 1	(R): Previous radiotherapy: For primary tumour = 2 For other disease = 1 None = 32	(R): Sphincter-preserving = 19 Non-sphincter-preserving = 16	35	CT/MRI	Regional anatomical (Hruby)^31^	Anastomotic = 5 Posterior = 18 Perineal = 7 Lateral = 5	N/A
Valentini *et al*. 1999^54^	Italy	8	Prospective	(R): 62	47	(R): 29M : 18F	Not reported	Not reported	(R): External beam RT: Neoadjuvant = 7 Adjuvant = 6 Adjuvant chemotherapy = 6	(R): LAR = 33 APER = 14	47	CT	Regional anatomical Modified Pilipshen (Memorial Sloan-Kettering^50^) and fixity (modified Mayo clinic^7^)	Anastomotic = 26 Pelvic = 21 F0 = 2 F1 = 11 F2 = 13 F3 = 18 F4 = 3	Patients with F0/F1 LR had 5-year survival rate of 100% compared with 0–14% in those with tumours graded F2+ (*P* < 0.008) and experiencing pain significantly correlated with the ‘F’ grading (*P* = 0.01)
Westberg *et al*. 2017^56^	Sweden	7	Retrospective	(TP): 72	149 ^$^	(R): 80M : 69F	Stage (R): I = 26 II = 52 III = 68 Unknown = 3	(R): N-ve = 78 N+ve = 68 Unknown = 3	(R): Neoadjuvant treatment: None = 93 Chemoradiotherapy = 56	(R): APER = 26 Hartmann’s = 16 LAR = 107	149	CT/MRI	Regional anatomical – combination of Leeds^21^ and Memorial Sloan-Kettering^10^	Central = 89 Non-central = 60	Significant increase in death of patients with LR in ‘non-central’ pelvic location (*P* = 0.014)
Yamada *et al*. 2001^8^	Japan	16	Retrospective	Not reported	60	(R): 38M : 22F	Dukes (R): A = 7 B = 16 C = 37	(R): N-ve = 23 N+ve = 37	(R): Most patients received adjuvant chemotherapy Adjuvant radiotherapy = 0	(R): APER = 28 Sphincter-sparing = 32	60	CT/MRI/abdominal ultrasound/EUS	Regional anatomical (Yamada *et al*.^8^)	Localized = 27 Sacral invasive = 16 Lateral invasive = 17	Significant difference in 5-year survival rates according to pattern of pelvic invasion following surgery for LRRC: 0 *versus* 10 *versus* 38% for those with lateral invasive *versus* sacral invasive *versus* localized invasion, respectively
Yun *et al*. 2016^61^	Korea	14	Retrospective	(R): 58	2050	(TP): 1233M : 817F (R): 84M : 63F	TNM (R): T0 = 2 T1 = 3 T2 = 22 T3 = 111 T4 = 9	(R): N0 = 62 N1 = 44 N2 = 41	(R): Neoadjuvant treatment CCRT = 29 Adjuvant chemotherapy Yes = 84 No = 34 Adjuvant radiotherapy: Yes = 67 No = 51	(R): TME–sphincter-preserving = 108 Non-sphincter-preserving = 39	147	PET CT/CT/MRI/EUS	Regional anatomical (Kusters *et al*.^14^)	Anterior = 7 Posterior = 29 Lateral = 52 Anastomotic = 48 Perineal = 11	Amalgamated categories within Kusters system into axial and non-axial, reporting that the site of LR did not affect subsequent prognosis (*P* = 0.146)
Zhao *et al*. 2012^62^	Japan	8	Retrospective	(R): 59.4	1079	(R): 54M : 36F	Stage (R): I = 6 IIA = 8 IIB = 11 IIIA = 10 IIIB = 20 IIIC = 26 Unknown = 9	(R): N-ve = 25 N+ve = 56 Unknown = 9	(R): Neoadjuvant chemoradiotherapy Yes = 18 No = 72	(R): APER = 32 AR = 54 Hartmann's = 2 LE = 2	79	CT/MRI	Regional anatomical (Memorial Sloan-Kettering^10^)	Axial = 27 Anterior = 21 Posterior = 8 Lateral = 23	Resectability maximal in axial tumours compared to lateral tumours, 88.9 *versus* 21.7% respectively (*P* < 0.001) Location of LR had a significant impact on R0 resection: axial = 85.2%, anterior = 33.3%, posterior = 25% and lateral = 4.3% (*P* < 0.001)
Zhu *et al*. 2016^63^	Japan	5	Retrospective	(R): 56	135	(R): 73M : 62F	TNM (R): T1 = 2 T2 = 35 T3 = 68 T4 = 30	(R): N0 = 43 N1 = 62 N2 = 30	(R): Radiotherapy: Neoadjuvant = 19 Adjuvant = 6	(R): APER = 78 LAR = 57	135	PET CT/CT/MRI/EUS	Regional anatomical based on Kusters *et al*.^14^ and Memorial Sloan-Kettering^10^	Presacral = 33 Lateral = 30 Anterior = 26 Anastomosis = 31 Perineum = 7 Internal iliac lymph node = 8	Patients with anastomotic LR had superior 5-year survival rate of 80.5% compared with 57.7% *versus* 44.5% for anterior *versus* ‘other’ LR respectively (*P* = 0.037)

* Japanese patients only included here as Dutch TME trial patients included below.

† Data unavailable for all patients.

‡ Includes 27 patients who had R1 resection at primary surgery. APR/APER, abdominoperineal excision of rectum; AR, anterior resection; CAPR, combined abdominoperineal resection; CCRT, concurrent chemoradiotherapy; DFS, disease-free survival; EUS, endoscopic ultrasound; HAR, high anterior resection; IORT, Intra-operative radiotherapy; JSCCR, Japanese Society for Cancer of the Colon and Rectum; Lap, laparoscopic; LAR, low anterior resection; LE, local excision; LLND, lateral lymph node dissection; LR, local recurrence; N/A, not applicable; pCR, pathological complete response; PE, pelvic exenteration; PSD, pelvic sidewall dissection; R, recurrences; RT, radiotherapy; TAE, transanal excision; TaTME, transanal TME; TME, total mesorectal excision; TP, total population; TRUS, transrectal ultrasound; UICC, Union for International Cancer Control.

These 21 studies, including six prospective and 15 retrospective series, comprised 2086 patients who developed LR following surgery for primary rectal/rectosigmoid adenocarcinoma. One study also included 19 patients following surgery for sigmoid colon adenocarcinoma^32^. LRs within these studies were classified according to a previously defined system proposed by institutions or institutional groups (Mayo Clinic, Memorial Sloan-Kettering – original and modified, Royal Marsden and Leeds) or authors (Yamada, Hruby and Kusters), and are outlined in *Table 2*^7,8,10–13,15,38^. Each system describes LR either according to a compartmentalized anatomical site of pelvic invasion, examples including axial, central, lateral or posterior; the presence or absence of associated symptoms; or, finally, fixation to adjacent structures within the pelvis. Within each study these categorizations have enabled evaluation of oncological outcomes, predominantly resection (R) status and prognostic information according to the sites of LR. *Table 3* summarizes the relevant results from each of the eight defined classification systems in use, focusing on prediction of R0 resection and survival outcomes.

**Table 2:** Defined classification systems included

Study group	Classification	Definition
Mayo Clinic^7^	Symptoms Fixation to surrounding structures (within the pelvis)	**S0:** asymptomatic **S1:** symptomatic without pain **S2:** symptomatic with pain **F0:** no sites of fixation **F1:** 1 site of fixation **F2:** 2 sites of fixation **F3:** 3 or 4 sites of fixation
Yamada *et al*.^8^	Pattern of pelvic invasion	**Localized:** tumour localized to adjacent pelvic organs **Sacral invasive:** tumour invades the lower sacrum (S3/S4/S5), coccyx or periosteum **Lateral invasive:** tumour invades the sciatic nerve, greater sciatic foramen, lateral pelvic sidewall or upper sacrum (S1/S2)
Memorial Sloan-Kettering Updated (Moore *et al*.^10^)	Tumour involvement (often ≥1 compartment)	**Axial:** tumour not involving anterior/posterior/lateral pelvic walls, e.g., anastomotic/perineal recurrence/mesorectum **Anterior:** tumour involving urinary bladder, vagina, uterus, seminal vesicles or prostate **Posterior:** tumour involving the sacrum or coccyx **Lateral:** tumour involving the bony pelvic sidewall or its structures including: iliac vessels/pelvic ureters/lateral lymph nodes/pelvic autonomic nerves/sidewall musculature
Royal Marsden group^11^	Pattern of pelvic invasion (structures within each compartment)	**Anterior above peritoneal reflection:** ureters, iliac vessels above peritoneal reflection, sigmoid colon, small bowel, lateral pelvic sidewall fascia (peritoneal surface) **Anterior below peritoneal reflection:** genitourinary system (seminal vesicles, prostate, uterus, vagina, ovaries, bladder/vesicoureteric junction, proximal urethra), pubic symphysis **Central:** rectum/neo-rectum (intra/extraluminal), perirectal fat or mesorectal recurrence **Posterior:** coccyx, presacral fascia, retrosacral space, sacrum, sciatic nerve, sciatic notch, S1 and S2 nerve roots **Lateral:** internal and external iliac vessels, lateral pelvic lymph nodes, piriformis muscle, internal obturator muscle **Infralevator:** levator ani muscles, external sphincter complex, ischioanal fossa **Anterior urogenital triangle:** perineal body/perineal scar (if previous abdominoperineal resection of rectum), vaginal introitus, distal urethra, crus penis
Leeds group^12^	Pattern of pelvic invasion	**Central:** tumour confined to pelvic organs or connective tissue without contact onto, or invasion into, bone **Sacral:** tumour present in the presacral space and abuts onto or invades the sacrum **Sidewall:** tumour involving lateral pelvic sidewall structures including greater sciatic foramen and sciatic nerve through to piriformis and the gluteal region **Composite:** sacral and sidewall combined
*Hruby et al.* ^13^	Pattern of pelvic invasion	**Anterior pelvic:** anterior pelvic organs including bladder/prostate/vagina **Posterior central**: including presacral space **Pelvic sidewall** **Anastomotic:** involving/abutting the anastomosis **Perineal**
Kusters *et al*.^14^	Pattern of pelvic invasion	**Presacral:** predominantly midline, in contact with sacral bone **Anterior:** predominantly midline, involving bladder/uterus/vagina/seminal vesicles or prostate **Anastomotic:** recurrence after low anterior resection or Hartmann’s procedure at the staple line **Lateral:** pelvic sidewall, immediately behind posterior ischial spine, in the obturator lymph node compartment or along iliac vessels **Perineal:** anal sphincter complex with surrounding perianal and ischiorectal space
Memorial Sloan-Kettering Original (Pilipshen *et al*.^15^)	Pattern of pelvic invasion	**Anastomotic:** a suture-line local recurrence with histological verification and no clinically apparent contiguous extramural disease **Perianastomotic:** limited extramural recurrence at the approximate level of the anastomosis without pelvic fixation, i.e., potentially resectable **Pelvic disease with sacral or sidewall and anterior fixation** precluding resection **Pelvic disease** (with or without fixation) presenting through the anastomosis

**Table 3:** Summary of outcomes

Study group	Studies using this classification system	Summary of results
Mayo Clinic^7^	Suzuki *et al*.^7^ Boyle *et al*.^12^ Hahnloser *et al*.^30^ Valentini *et al*.^54^	Increasing risk of severe complications with increasing degree of fixation – F0 = 14% *versus* F3 = 44%^7^ Following surgery for LRRC 37% of patients with F0/F1 LR suffered postoperative complications *versus* 54.5% for F2 disease^12^. Surgical complication rates significantly associated with the number of sites of fixation – 20% in F0/F1 tumours, 35% in F2 tumours and 32% in F3+ tumours (*P* = 0.05)^30^ 3- and 5-year survival rates: S0/S1patients, 68.4 and 37.3%; S2 patients, 31.6 and 26.3%^7^ 3- and 5-year survival rates: F0 patients, 61.3 and 50%, F1–F3, 35.7 and 31.2%^7^ Increasing points of pelvic fixation significantly reduced survival at both 3 and 5 years (*P* < 0.0001)^30^ F0/F1 LR – 5-year survival rate 100% *versus* 0–14% in F2+LR (*P* < 0.008). Experiencing pain was significantly correlated with the ‘F’ grading (*P* = 0.01)^54^
Yamada *et al*.^8^	Yamada *et al*.^8^ Bird *et al*.^22^ Kanemitsu *et al*.^33^	5-year survival rates: 0 *versus* 10 *versus* 38% for those with lateral invasive *versus* sacral invasive *versus* localized invasion, respectively^8^ Poorer progression-free survival in patients with lateral invasive or sacral invasive LR (*P* < 0.05)^22^ Pattern of pelvic invasion affected the likelihood of R0 resection (*P* = 0.005) and local disease-free survival following surgery for LR (*P* = 0.028)^33^
Memorial Sloan-Kettering^10^^,^^15^	Moore *et al*.^10^ Pilipshen *et al*.^15^ Iversen *et al*.^32^ Sinaei *et al*.^51^ Valentini *et al*.^54^ Westberg *et al*.^56^ Zhao *et al*.^62^ Zhu *et al*.^63^	Pelvic sidewall involvement demonstrated on imaging – R0 resection in 60% of patients^10^ Axial compartment alone occupied by tumour intraoperatively – R0 resection rate of 70 *versus* 43% when other compartments involved (*P* < 0.001)^10^ Axial and anterior compartments both occupied by recurrent tumour – R0 resection in 72 *versus* 42% when tumour occupied other intrapelvic compartments (*P* = 0.003)^10^ R0 resection rate greater if lateral compartment not involved intraoperatively in comparison to an involved lateral compartment (65 *versus* 36%, *P* = 0.002)^10^ also reported by Iversen *et al*. (90 *versus* 63%, *P* = 0.004)^32^ Iliac vessel involvement – R0 resection rate 17 *versus* 55% when not involved (*P* = 0.01)^10^ Resectability maximal in axial tumours *versus* lateral tumours, 88.9 *versus* 21.7% respectively (*P* < 0.001)^62^ Location of recurrent tumour had a significant impact on R0 resection rate: axial = 85.2%, anterior = 33.3%, posterior = 25% and lateral = 4.3% (*P* < 0.001)^62^
Royal Marsden Group^11^	Roodbeen *et al*.^67^	Poorer overall survival if tumour within the ‘anterior above peritoneal reflection’ compartment on MRI *versus* if this compartment not involved (*P* = 0.012)^11^ Patients with tumour within the lateral and posterior compartments, or within three or more compartments had a reduced disease-free survival^69^
Leeds Group^12^	Boyle *et al*.^12^ Westberg *et al*.^56^	LR in a ‘non-central’ pelvic location – significant increase in death (*P* = 0.014)^56^
Hruby *et al*.^13^	Hruby *et al*.^13^ Uehara *et al*.^53^	Primary T4 rectal cancers most frequently recurred in the anterior central compartment (*P* < 0.01)^13^ LR following an APER most frequent in perineal location (*P* < 0.01)^13^
Kusters *et al*.^14^	Kusters *et al*.^14^^,^^37^^,^^38^ Yun *et al*.^61^ Zhu *et al*.^63^	5-year LR rate in anterior compartment: TME + radiotherapy for primary rectal adenocarcinoma 0.7 *versus* 2.7% in patients undergoing TME surgery alone (*P* = 0.003)^61^ APER for primary rectal adenocarcinoma – 5-year LR rate 11.7%, usually occurring in the presacral compartment (45%)^38^ LAR 5-year LR rate 7.8% usually resulted in anastomotic (36%) and presacral (28%) LR^38^ The site of LR did not affect subsequent prognosis (*P* = 0.146)^61^ Patients with ‘anastomotic’ LR – 5-year survival rate 80.5% *versus* 57.7% *versus* 44.5% for anterior *versus* ‘other’ LR respectively (*P* = 0.037)^63^

LR, local recurrence; APER, abdominoperineal excision of rectum; TME, total mesorectal excision; LAR, low anterior resection.

### Mayo Clinic

The Mayo Clinic system was used in four studies to report on LR^7^^,^^12^^,^^30^^,^^54^, with two of these studies combining an additional anatomical classification. The Mayo Clinic system classifies LR according to the degree of fixation to surrounding structures within the pelvis and symptoms associated with the recurrence^7^^,^^70^. Fixity is graded from no sites of fixation, F0, to F3 (3 to 4 sites of fixation) and from asymptomatic (S0), to symptoms of LR including pain (S2). Using this system, it has been demonstrated that patients undergoing surgery for LRRC had an increasing risk of severe complications as the degree of fixation increased, from 14 per cent in F0 patients, to 44 per cent in F3 patients^7^. The Leeds group slightly modified the classification system, where an F2 grade represented tumour fixation at two or more sites. When reporting on patients following surgery for LRRC, using this modified system, 37 per cent of patients with F0/F1 LR suffered postoperative complications compared to 54.5 per cent in those with F2 disease^12^. Survival rates were also shown to be impacted by pelvic fixation and symptoms. The 3- and 5-year survival rates were 68.4 and 37.3 per cent respectively for patients without pain (S0/S1), compared with 31.6 and 26.3 per cent respectively for those with pain (S2). The 3- and 5-year survival rates were 61.3 and 50 per cent respectively for those patients with no disease fixation (F0) and 35.7 and 31.2 per cent respectively for those with some degree of disease fixation (F1–3)^7^. It was demonstrated that surgical complication rates were significantly associated with the number of sites of fixation of the locally recurrent tumour, 20 per cent in those with F0/F1 tumours, 35 per cent in F2 tumours and 32 per cent in F3+ tumours (*P* = 0.050). The same study also affirmed that increasing the number of points of pelvic fixation significantly reduced survival at both 3 and 5 years (*P* < 0.0001)^30^. Another author also modified the Mayo Clinic system as follows: F0, no evidence of contact with the pelvic sidewall; F1, extent of contact less than quarter of the pelvic sidewall; F2, contact extends to between quarter and half of the circumference of the pelvic sidewall; F3, contact with more than half of the circumference of the pelvic sidewall; and F4, infiltration of bony structures or the small bowel^54^. This author reported that patients with F0/F1 LR tumours had a 5-year survival rate of 100 per cent compared with 0–14 per cent in those with tumours graded F2+ (*P* < 0.008) and that experiencing pain was significantly correlated with the ‘F’ grading (*P* = 0.01)^54^.

### Yamada and colleagues

Out of 21 studies, three studies^8^^,^^22^^,^^33^ used the Yamada system^8^ to report on LR. This categorizes LR according to the pattern of invasion within the pelvis: local, lateral invasive or sacral invasive. Another author when reporting LR used this system, but in addition, documented the anatomical ‘site’ of LR^22^. Another modification of Yamada classification was further proposed, dividing the level of sacral invasion into two compartments and classifying anastomotic recurrence as a separate entity^33^. Yamada and colleagues demonstrated a significant difference in 5-year survival rates according to the pattern of pelvic invasion following surgery for LRRC. The following 5-year survival rates were observed: 0 *versus* 10 *versus* 38 per cent for those with lateral invasive *versus* sacral invasive *versus* localized invasion, respectively^8^. This was validated and a poorer progression-free survival in patients with lateral invasive or sacral invasive LRs (*P* < 0.05) was also reported^22^. In a different experience, the pattern of pelvic invasion affected the likelihood of R0 resection (*P* = 0.005) and local disease-free survival following surgery for LR (*P* = 0.028)^33^.

### Memorial Sloan-Kettering (original and modified)

Pilipshen from the Memorial Sloan-Kettering group first described a classification system for LRRC in 1984, which was later refined by Moore 20 years later, categorizing tumour involvement into intrapelvic compartments: axial, anterior, posterior or lateral. The Memorial Sloan-Kettering classifications were used in eight studies^10^^,^^15^^,^^32^^,^^51^^,^^54^^,^^56^^,^^62^^,^^63^ when reporting LR. The modified classification established that if the pelvic sidewall was not involved by recurrent tumour on imaging, this resulted in R0 resection in 60 per cent of patients. When the axial compartment alone was occupied by tumour intraoperatively, this resulted in an R0 resection rate of 70 per cent, compared with 43 per cent when other compartments were involved (*P* < 0.001). When both the axial and anterior compartments were occupied by recurrent tumour, this resulted in R0 resection in 72 per cent compared with 42 per cent when tumour occupied other intrapelvic compartments (*P* = 0.003)^10^. The rate of R0 resection was greater if the lateral compartment was not involved intraoperatively in comparison with patients with an involved lateral compartment (65 *versus* 36 per cent, *P* = 0.002), which was also reported by Iversen and co-workers (90 *versus* 63 per cent, *P* = 0.004)^32^. Finally, involvement of the iliac vessels resulted in R0 resection in 17 per cent, compared with 55 per cent when the iliac vessels were not involved (*P* = 0.01)^10^. Another manuscript remarked on ‘resectability’ of a tumour dependent on its pelvic location. It was reported that resectability was maximal in axial tumours compared with lateral tumours, 88.9 *versus* 21.7 per cent respectively (*P* < 0.001), demonstrating also that the location of recurrent tumour within the pelvis also had a significant impact on R0 resection: axial, 85.2 per cent; anterior, 33.3 per cent; posterior, 25 per cent; and lateral, 4.3 per cent (*P* < 0.001)^62^.

### Royal Marsden group

**Fig. 2:**
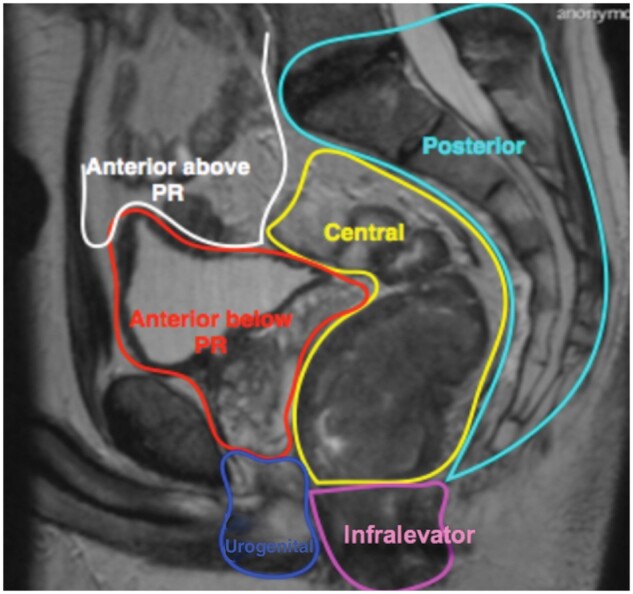
MRI sagittal view of defined Royal Marsden group intrapelvic compartments. PR = peritoneal reflection

**Fig. 3:**
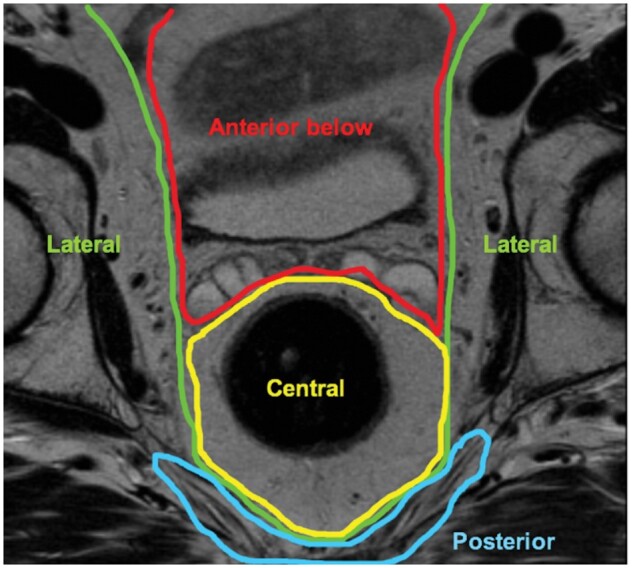
MRI axial view of defined Royal Marsden group intrapelvic compartments PR, peritoneal reflection

There was a single assessable study^67^ using the Royal Marsden system to report on LR. This classification divides the pelvis into seven compartments according to fascial boundaries: central, anterior above the peritoneal reflection, anterior below the peritoneal reflection, posterior, lateral, infralevator and anterior urogenital triangle (*Figs 2* and *3*). The Royal Marsden classification was the single system within this review accompanied by an illustration of the pelvic compartments^11^. Use of this system has demonstrated that patients with tumour within the ‘anterior above peritoneal reflection’ compartment on MRI had a poorer overall survival compared with patients where this compartment was not involved (*P* = 0.012)^11^. It was also reported that patients with tumour within the lateral and posterior compartments, or within three or more compartments, had a reduced disease-free survival^69^.

### Leeds group

This system, classifying tumour within the pelvis according to the patterns of pelvic invasion (central, sacral, sidewall and composite (sidewall and sacral combined)), was implemented by two studies to report on LR^12^^,^^56^. The most recent, in 2017, combined the Memorial Sloan-Kettering system along with the Leeds group system to report on LR within the categories of: axial/anterior (central), posterior/lateral (posterolateral) and multifocal, demonstrating a significant increase in death of patients whose LR was in a ‘non-central’ pelvic location (*P* = 0.014)^56^. The Leeds group did not report on LRs or outcomes using their system alone, but in conjunction with the Mayo clinic classification, as reviewed above, in relation to tumour fixation.

### Hruby and colleagues

Hruby and colleagues categorize the pelvis into five compartments: anterior pelvic, posterior central, anastomotic, pelvic sidewall and perineal, and this system was used in two studies to report on LR^13^^,^^53^. These compartment categories were revised in 2015, however the two systems are largely similar.

Hruby and co-workers did not demonstrate any significant effect on survival dependent on the location of LR, but that primary T4 rectal cancers most frequently recurred in the anterior central compartment (*P* < 0.01) and that abdominoperineal excision of the rectum (APER) resulted most commonly in perineal LR (*P* < 0.01)^13^.

### Kusters and colleagues

This system, compartmentalizing the pelvic regions into presacral, anastomotic, anterior, lateral and perineal involvement, was used in five studies^14^^,^^37^^,^^38^^,^^61^^,^^63^. The system was implemented in the Dutch TME trial in over 1400 patients to report on LR. They demonstrated that patients undergoing TME with radiotherapy for primary rectal adenocarcinoma had a 5-year LR rate of 0.7 per cent in the anterior compartment compared with 2.7 per cent in those patients undergoing TME surgery alone (*P* = 0.003). It was also reported that patients undergoing APER for primary rectal adenocarcinoma had a 5-year LR rate of 11.7 per cent, usually occurring in the presacral compartment (45 per cent), compared with a 5-year LR rate of 7.8 per cent in those undergoing low anterior resection (LAR), which usually resulted in anastomotic (36 per cent) and presacral (28 per cent) LR^38^. Another author amalgamated categories within this system into axial and non-axial LR and reported that the site of LR did not affect subsequent prognosis (*P* = 0.146)^61^. In a different report, authors also modified this system to combine anastomotic and perineal recurrences with a separate category for ‘lymph node’ LR. They showed that patients with ‘anastomotic’ LR had a superior 5-year survival rate of 80.5 per cent compared with 57.7 *versus* 44.5 per cent for anterior *versus* ‘other’ LR respectively (*P* = 0.037)^63^.

## Discussion

There is currently no single standardized classification system used to describe LRRC, however, the systems reviewed provide valuable information focusing on three main areas: disease ‘extent’ within the pelvis, symptoms associated with LR and finally more detailed anatomical information on disease location. The majority of classification systems have not been validated preoperatively against oncological outcomes. Describing pelvic LR is based predominantly on the anatomical location and therefore aetiology of the recurrence. Consequently, imaging is the only method of defining and describing recurrences objectively. Although MRI is the optimal imaging modality for the assessment of LR^71^, this was stated as the main diagnostic tool in only five evaluated studies; however, many of these classification systems were described prior to the development of, widespread use of and increasing accuracy of MRI.

The Mayo Clinic system provides an indication of disease extent by outlining the number of points of fixation within the pelvis, and consequently its use has provided beneficial prognostic information that can assist decision making regarding treatment, for example the required surgical procedure or neoadjuvant therapy. Experienced symptoms are also suggestive of disease burden, however this is less specific, as pain may not be experienced unless tumour is involving adjacent nerves. The system is limited in that no anatomical detail regarding tumour site is specified.

The system outlined by Yamada and colleagues provides more general information on LR, with sacral and lateral invasion being self-explanatory as more advanced pathology. A ‘localised’ tumour is non-specific and not indicative of which ‘adjacent’ pelvic organs are involved or may require resection. Involvement of the posterior prostatic wall *versus* the anal sphincter complex, may have significantly different consequences for the patient. Implementing this system alone, without the precise location of LR, would make targeting perioperative radiotherapy and surgical planning considerably challenging.

The Memorial Sloan-Kettering, Royal Marsden, Kusters *et al*. and Hruby *et al*. systems are somewhat similar and provide detailed information on the tumour with regards to its location in relation to surrounding pelvic viscera. This is informative for the operating surgeon as an indication of potential structures which may require resection, and also for the oncologist as to which areas may require targeting with radiotherapy. The Royal Marsden system separates pelvic compartments along fascial boundaries and therefore highlights the anatomical planes required to be entered, or excised, in order to remove the tumour. This is the most detailed anatomical system within the studies reviewed, categorizing LR ‘above the peritoneal reflection’ as a separate anterior entity, and tumour within this compartment was previously shown to have poorer survival outcomes^11^. The Kusters *et al*. system, which is based on the same boundaries as described by Roels *et al*.^72^, also divides the pelvis according to its fascial boundaries but anterior structures above and below the peritoneal reflection are encompassed within the same compartment, and the inferior structures (levator muscles, anal-sphincter complex, ischiorectal fossa and perineum) are also within another single compartment^72^.

Memorial Sloan-Kettering does not specify the boundaries of each compartment and therefore tumour assessment intraoperatively may be difficult if not directly involving or in between structures^10^.

The Leeds group system is an informative system as, like the Mayo Clinic and Yamada *et al*. systems, it focuses on the pattern of more advanced tumour invasion within the pelvis. Central involvement is non-specific as to which anterior pelvic viscera may be involved or require resection, but the system highlights sacral and lateral involvement, which are potentially more problematic tumours to treat. As the Leeds group classification system was not used to report on outcomes other than in conjunction with the Mayo Clinic system, it is difficult to quantify its prognostic or operative benefit.

A limitation of this review and introduction of potential selection bias, is that, although the method of diagnosing LR is stated usually as a combination of imaging, biochemical tests and endoscopy, the method of classification is not implicitly stated in the majority of studies. Therefore, outcomes may differ dependent on the imaging method used (i.e., CT/MRI). An additional source of potential selection bias is that some of the study cohorts were patients with ‘advanced’ T3+ primary tumours only, whereas other studies did not select for T-stage.

R0 resection is the best predictor of survival in patients with LRRC^2^ and currently improvements in R0 resection rates are largely attributed to optimal preoperative imaging in surgical planning. This facilitates appropriate preoperative therapy, planning radicality of an operation and selecting out patients unlikely to benefit from pelvic exenteration. Uniformity of the language used to describe LR and its classification is required to optimize R0 resection rates and subsequently provide prognostic information to patients in the future. Each defined classification system, as discussed in this review, has potential benefits and a standardized system would enable oncological and survival outcomes to be compared internationally, improving the standard of care for patients with this pathology. Each system has a distinctive method, and terminology, for describing LR and, as a result of the outcomes illustrated, standard surgical techniques may be reconsidered, for example, modification of resection margins.

In order to predict the likelihood of R0 resection correctly in this complex cohort of patients it is therefore important to use the gold standard technique of MRI to classify LR and ensure accurate assessment of the intrapelvic structures. CT and PET-CT are also important, to be used in conjunction with MRI, most often to try to exclude distant metastases. This is fundamental in the overall assessment of the patient and as an indicator of available treatment options, if appropriate. Whatever the adopted system, maximizing the anatomical detail provided by the imaging assessing recurrence, will optimize therapeutic planning and oncological outcomes.

*Disclosure*. The authors declare no conflict of interest.

## Supplementary material

Supplementary material is available at *BJS Open* online.

## Supplementary Material

zrab024_Supplementary_Data
