# Supplementary material for: Systematic review of classification systems for locally recurrent rectal cancer
Source: BJS Open. 2021 Apr 8;5(3):zrab024. doi: 10.1093/bjsopen/zrab024 (PMC8105621; doi:10.1093/bjsopen/zrab024)
Supplement: zrab024_Supplementary_Data [file zrab024_supplementary_data.docx]

**Supplementary Table 1: Detailed Search Strategy**

| **Database** | **Time span** | **Search strategy** | |
| --- | --- | --- | --- |
| Cochrane Central Register of Controlled Trials (CENTRAL) in The Cochrane Library (Wiley) | April 28^th^ 2020 | #1 rectal cancer  #2 local recurrence  #3 patterns OR classification OR risk factors  #4 (#1 AND #2 AND #3) | |
| MEDLINE (Pubmed) | January 1947 to  April 28^th^ 2020 | (rectal cancer) AND (local recurrence) AND (patterns OR classification OR risk factors) | |
| EMBASE (OvidSP) | January 1947 to  April 28^th^ 2020 | 1 (rectal cancer).af.  2 (local recurrence).af.  3 (patterns OR classification OR risk factors).af  4 1 AND 2 AND 3 | |
| Science Citation Index Expanded (http://www.webofknowledge.com/?DestApp=WOS) | January 1970 to  April 28^th^ 2020 | #1 TS=(rectal cancer)  #2 TS=(local recurrence)  #3 TS=(patterns OR classification OR risk factors)  #4 #1 AND #2 AND #3 |  |
